# Viral Infections in Elderly Individuals: A Comprehensive Overview of SARS-CoV-2 and Influenza Susceptibility, Pathogenesis, and Clinical Treatment Strategies

**DOI:** 10.3390/vaccines13040431

**Published:** 2025-04-21

**Authors:** Yanhao Huang, Shumin Li, Wenjie Ye, Haoyun Wang, Jun Su, Lijuan Gao, Ruohu Shi, Xinyi Mou, Sean Xiao Leng, Chanchan Xiao, Guobing Chen

**Affiliations:** 1 The Sixth Affiliated Hospital of Jinan University (Dongguan Eastern Central Hospital), School of Medicine, Jinan University, Dongguan 523000, China; yhhuang@stu2020.jnu.edu.cn; 2 Department of Microbiology and Immunology, Institute of Geriatric Immunology, School of Medicine, Jinan University, Guangzhou 510632, China; shuminli1999@163.com (S.L.); wenjie@stu2024.jnu.edu.cn (W.Y.); why713012@163.com (H.W.); gaolijuan@jnu.edu.cn (L.G.); shiruohu@163.com (R.S.); muxinyi@stu2022.jnu.edu.cn (X.M.); 3 Key Laboratory of Viral Pathogenesis & Infection Prevention and Control (Jinan University), Ministry of Education, Guangzhou 510632, China; 4 Guangdong-Hong Kong-Macau Great Bay Area Geroscience Joint Laboratory, School of Medicine, Jinan University, Guangzhou 510632, China; 5 First Affiliated Hospital, Jinan University, Guangzhou 510632, China; junsu@jnu.edu.cn; 6 Johns Hopkins Center on Aging and Immune Remodeling, Division of Geriatric Medicine and Gerontology, Departments of Medicine, Molecular Microbiology and Immunology, Johns Hopkins University School of Medicine and Bloomberg School of Public Health, Baltimore, MD 21205, USA; sleng1@jhu.edu; 7 Zhuhai Institute of Jinan University, Jinan University, Zhuhai 519070, China

**Keywords:** aging, SARS-CoV-2, influenza viruses, coinfections, pathogenesis

## Abstract

As age increases, the immune function of elderly individuals gradually decreases, increasing their susceptibility to infectious diseases. Therefore, further research on common viral infections in the elderly population, especially severe acute respiratory syndrome coronavirus 2 (SARS-CoV-2) and influenza viruses, is crucial for scientific progress. This review delves into the genetic structure, infection mechanisms, and impact of coinfections with these two viruses and provides a detailed analysis of the reasons for the increased susceptibility of elderly individuals to dual viral infections. We evaluated the clinical manifestations in elderly individuals following coinfections, including complications in the respiratory, gastrointestinal, nervous, and cardiovascular systems. Ultimately, we have summarized the current strategies for the prevention, diagnosis, and treatment of SARS-CoV-2 and influenza coinfections in older adults. Through these studies, we aim to reduce the risk of dual infections in elderly individuals and provide a scientific basis for the prevention, diagnosis, and treatment of age-related viral diseases, thereby improving their health status.

## 1. Introduction

**Figure 1:**
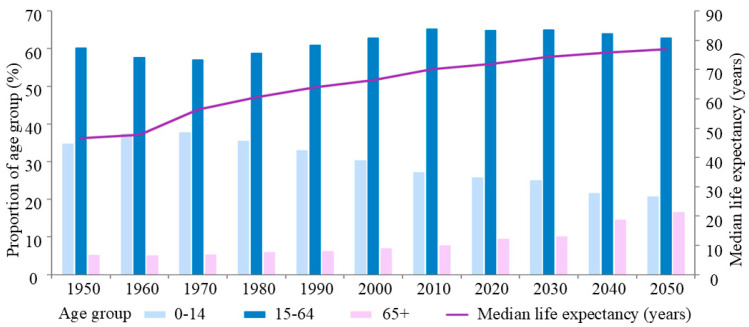
World population age structure distribution from 1950 to 2050. Changes in the proportions of three age groups (0–14, 15–64, and 65+) and the median life expectancy (years) worldwide from 1950 to 2050. Owing to factors such as societal development and shifts in public health perceptions, there has been a significant increase in median life expectancy. The proportion of the population aged 65 and over has been steadily rising over time, indicating a trend toward an aging global population structure. Data source: Population Division of the Department of Economic and Social Affairs of the United Nations. (see https://population.un.org/wpp (accessed on 2 September 2024) for more details).

The aging population has rapidly increased and has become a significant socioeconomic issue that is currently faced worldwide [1] (Figure 1). In China, it is expected that by 2050, there will be a population of 1.4 billion people, including 365 million people aged 65 and above, accounting for 26.1% of the total population in the country [2]. As age increases, human immune function changes, leading to a decreased ability to defend against external pathogens and clear mutated or senescent cells within the body. This makes the elderly more susceptible to various infectious and malignant diseases. Therefore, focusing on immune function and the aging of the immune system in elderly individuals is extremely important for promoting healthy aging within the population. Continuing to strengthen research on viral infections among elderly individuals holds profound significance for scientific advancement and social welfare.

Aging leads to a decline in immune function. The high incidence of influenza A viruses in spring and winter often overlaps with the COVID-19 pandemic, increasing the risk of illness in elderly individuals. Various studies have shown that the case fatality ratio (CFR) of COVID-19 increases with age, from 0.4% or lower in patients aged 40s or younger to 1.3% among those in their 50s, 3.6% in their 60s, 8% in their 70s, and 14.8% in their 80s or older [3]. Senior citizens also have a higher mortality rate after infection with the influenza virus. People aged 65 years or above have the highest hospitalization and mortality rates related to influenza [4]. A meta-analysis revealed that the all-cause mortality associated with influenza in China from 2005 to 2019 was 14.33 per 100,000 individuals, increasing to 122.79 per 100,000 individuals aged over 65 years [5]. Notably, coinfections of COVID-19 and influenza carry an increased risk of adverse clinical outcomes and a higher mortality rate, particularly among the elderly population [6,7]. In such cases of coinfection, the average age of hospitalized patients is significantly greater than that of nonhospitalized patients (42.1 ± 21 vs. 27.6 ± 20 years of age) [6]. The highest mortality rate is observed in patients over 65 years of age at 33.3%, followed by those aged 35–64 at 28.6% and those aged 15–34 at 11.1%, while no deaths have been reported among patients under 15 years of age [6].

The impact of viral diseases on an aging society continues to pose significant healthcare and socioeconomic challenges for policymakers and caregivers, highlighting the importance of elucidating the mechanisms behind the progression to severe illness in elderly individuals infected with single or coinfections of viral diseases. However, at present, there is a paucity of specific vaccines targeting SARS-CoV-2 and influenza coinfections in the elderly population. Furthermore, the diagnostic accuracy for this demographic remains suboptimal, and there is a lack of standardized treatment protocols tailored to this group. These factors may contribute to an increased rate of severe complications in elderly patients with coinfections.

In this review, we not only delve into the infection pathways and interactions of SARS-CoV-2 and influenza viruses within the human body but also thoroughly analyze the multiple factors that make the elderly more susceptible to severe illness, including immunosenescence, the presence of comorbidities, and the decline in physiological functions, as well as the specific clinical manifestations observed during this process. Through these studies, we aim to provide a scientific basis for the prevention and treatment of age-related viral infectious diseases and to develop targeted therapeutic strategies.

## 2. Results

### 2.1. Research Strategy

The following databases were investigated in an attempt to identify all the relevant studies published on PubMed, Web of Science, the Cochrane Library, EMBASE, and Google Scholar. The literature search was conducted up to 14 November 2024. The topic search terms used were as follows: #1—“SARS-CoV-2” OR “COVID-19”; #2—“influenza virus” OR “influenza viruses” OR “influenza”; #3—“coinfection” OR “codetection”; #4—“inflammaging” OR “immunosenescence” OR “aging”; #5—“manifestations” OR “clinical manifestations” OR “clinical outcome”; #6—“vaccine” OR “vaccination” OR “combination vaccine”; #7—#1 AND #2 AND #3; #8—#1 AND #2 AND #4; #9—#1 AND #2 AND #5; and #10—#1 AND #2 AND #6.

### 2.2. Eligibility Criteria

We included published articles that reported the proportion of SARS-CoV-2 and influenza virus coinfections. Only the studies published in English reporting SARS-CoV-2 infection confirmed by real-time reverse transcription–polymerase chain reaction (qRT–PCR) were included. Only the studies reporting influenza virus infection (A and/or B) by nucleic acid tests were included. Case reports, case series, and review articles were also included. Opinion articles and meta-analyses were also included.

To evaluate the mechanisms underlying coinfections with SARS-CoV-2 and influenza viruses, the genetic structures, infection mechanisms, and coinfection mechanisms of both viruses were investigated individually. To evaluate the role of aging in the coinfection of SARS-CoV-2 and influenza, the corresponding literature was individualized. To evaluate the clinical manifestations and complications of SARS-CoV-2 and influenza coinfection in older adults, subgroups (aged ≥ 65) were individualized.

### 2.3. Study Selection

**Figure 2:**
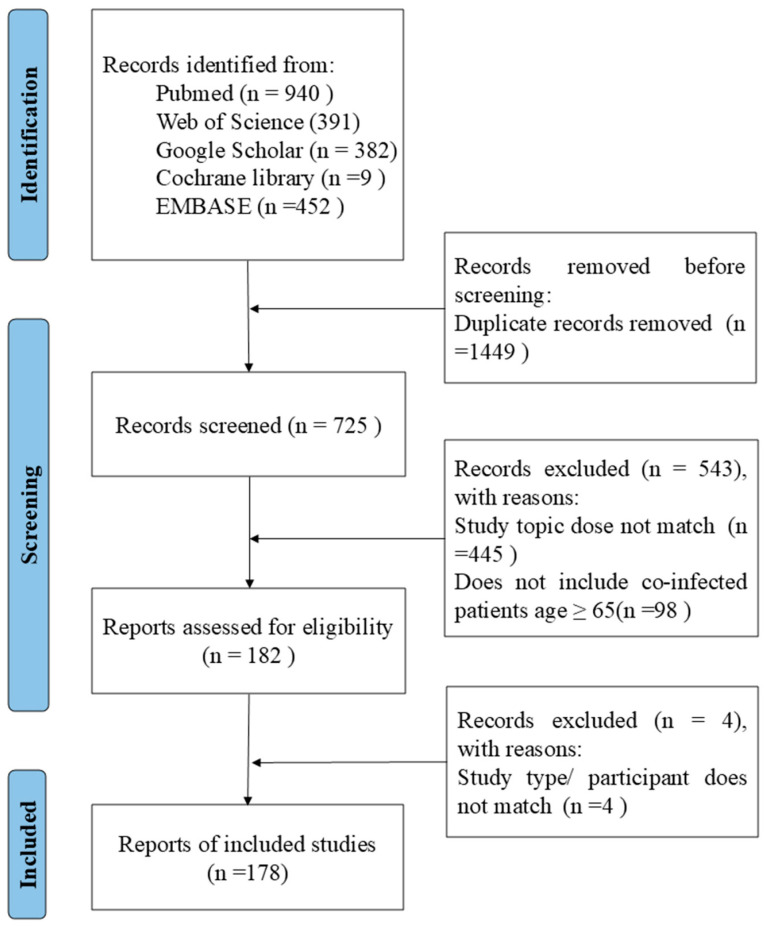
The process of study selection for older adults coinfected with SARS-CoV-2 and influenza.

After manually removing duplicates, the titles and abstracts of the articles identified through the initial search were first screened. The full texts of the relevant articles were examined for the inclusion and exclusion criteria (Figure 2). After screening the abstracts, the full texts of the articles were assessed for eligibility and were selected or rejected for inclusion in the review. Any discordant results were discussed in a consensus meeting.

## 3. Epidemiological Features and Coinfection Mechanisms of SARS-CoV-2 and Influenza

Since the outbreak of SARS-CoV-2 at the end of 2019, coinfection with the influenza virus has attracted widespread attention. Studies have shown that compared with patients with single viral infections, patients with coinfections have more severe conditions, with significantly increased risks of hospitalization and complications [6,7]. Although the genetic structure and infection mechanisms of the novel coronavirus and the influenza virus differ, the influenza virus can increase the infectivity of SARS-CoV-2 [8]. Coinfections lead to increased viral loads, immune cell infiltration, and elevated levels of inflammatory factors. In addition, coinfections also result in severe lymphocytopenia in the peripheral blood, reducing antibody levels and CD4^+^ T cell responses. With the arrival of the autumn and winter seasons, the risk of cocirculation and coinfection of the influenza virus and SARS-CoV-2 intensified, posing new challenges to public health [8,9]. Therefore, strengthening research on coinfection phenomena, improving vaccination rates, and implementing effective prevention and control measures are highly important for reducing the risk of coinfection.

### 3.1. Influenza Virus

Influenza viruses are a significant concern globally due to their ability to cause seasonal epidemics and occasional pandemics. The epidemiological features of these viruses vary widely depending on the type and subtype of the virus, geographical location, and population demographics. Influenza A and B viruses, officially known as *Alphainfuenzavirus infuenzae* and *Betainfuenzavirus infuenzae*, are primarily responsible for seasonal flu epidemics, with influenza A viruses being of particular concern due to their potential to cause pandemics. The H1N1 and H3N2 subtypes of influenza A continue to circulate in humans as seasonal influenza, contributing to significant morbidity and mortality worldwide [10]. WHO indicates that the influenza virus results in approximately 3 to 5 million severe infections and 290,000 to 650,000 fatal cases globally each year [11]. Influenza A virus (IAV) and influenza B virus (IBV) are extensively prevalent in human societies and often cause global pandemics [12]. IAV can be further divided into different subtypes on the basis of differences in surface glycoproteins—hemagglutinin (HA) and neuraminidase (NA)—with a total of 18 HA subtypes and 11 NA subtypes [13]. IBV does not distinguish subtypes but is divided into two lineages on the basis of differences in antigenicity and genetic characteristics (mainly based on HA): the B/Victoria/2/87-like lineage (B/Victoria) and the B/Yamagata/16/88-like lineage (B/Yamagata) [14].

The global pattern and determinants of interaction among seasonal influenza viruses reveal complex and heterogeneous interaction patterns. For instance, a consistent negative correlation exists between influenza A/H3N2 and A/H1N1 globally, with significant differences in interactions between influenza A and B across different countries. These interactions are primarily influenced by population-related factors, highlighting the need for targeted prevention strategies to reduce the global burden of seasonal influenza [15]. In China, the epidemiological and virological surveillance of influenza viruses during the 2020–2021 period showed a decline in influenza activity due to the COVID-19 pandemic. However, influenza activity began to recover in 2021, with the B/Victoria lineage being predominant. This resurgence underscores the importance of continuous surveillance and preparedness for co-epidemics of influenza and SARS-CoV-2. Similarly, another study in China during the same period found that influenza activity increased, although it remained below prepandemic levels. The majority of circulating viruses were B/Victoria lineage, emphasizing the need for strengthened influenza surveillance and vaccine recommendations [16].

These studies highlight the dynamic nature of influenza virus epidemiology and the importance of understanding the interactions and patterns of different influenza types and subtypes. Such insights are crucial for developing effective prevention and control strategies, including vaccination and public health interventions, to mitigate the impact of influenza globally.

#### Epidemiological Features of Influenza Viruses

Globally, influenza demonstrates seasonal outbreaks and increased incidence during the winter and spring seasons in temperate regions, while within tropical regions, particularly Asia, the seasonality of influenza is notably diverse, characterized by both annual periodicity, semiannual periodicity, and year-round periodicity [17,18,19,20]. A study conducted over four consecutive years surveilling influenza trends in China indicates that on average, A/H3N2 accounted for nearly half of the cases and circulated throughout all four seasons [20]. Meanwhile, A/H1N1, B/Victoria, and B/Yamagata exhibited peak activity during the winter and spring months, with a potential secondary peak observed in the southern regions during the summer season [20]. It is noteworthy that published data from 29 countries (1999–2014) show distinct age-related susceptibility to influenza strains: young children (<5 years) to A/H1N1, older children (5–17 years) to IBV, young adults (aged 18 to 39 years) and older adults (aged 40 to 64 years) to A/H1N1, and older people (aged ≥ 65 years) to A/H3N2 [21].

### 3.2. SARS-CoV-2

COVID-19 is caused by a new coronavirus known as SARS-CoV-2, which has a single-stranded RNA structure. The official name of the SARS-CoV-2 is *Betacoronavirus pandemicum*. As the virus replicates widely, SARS-CoV-2 continuously mutates, giving rise to multiple new variants. The current most dominant variants are BA.2.86, XBB.1.5, XBB.1.16, EG.5, and JN.1 (https://data.who.int/). Numerous studies indicate that there is no significant difference in symptoms caused by variants of concern (VOCs) and the wild type, but the intensity of symptoms varies significantly on the basis of the differences caused by different mutation sites, including transmission, pathogenicity, and immune escape ability [22,23].

#### Epidemiological Features of SARS-CoV-2

The epidemiological features of SARS-CoV-2 have been extensively studied to understand its transmission dynamics, mutation patterns, and impact on public health. The emergence of various SARS-CoV-2 variants has significantly influenced the global response to the COVID-19 pandemic. These variants, such as Alpha, Beta, Gamma, Delta, and Omicron, have shown increased transmissibility and potential resistance to neutralizing antibodies, which complicates efforts in epidemiological tracking and vaccine development [24].

In the context of specific regions, studies have highlighted the epidemiological characteristics of SARS-CoV-2 variants. For instance, the emergence of the Omicron variant in the southeast Brazilian population was marked by a significant increase in infection rates, particularly among younger age groups. This variant’s spread was characterized by specific genomic features that facilitated its transmission [25]. Similarly, in Japan, the molecular epidemiological features of SARS-CoV-2 were analyzed over multiple epidemic waves, revealing sharp displacements of lineages and genotypes, which provided insights into the virus’s genetic diversity and evolution [26].

A study that collected COVID-19 cases from the United States and Europe between March 2020 and December 2022 has indicated that although SARS-CoV-2 circulates throughout the year, seasonal epidemic peaks occur roughly from November to April, that is, during the winter and spring months [27]. During these months, an additional 75 hospitalizations and two deaths per million were observed [27]. Additionally, a study from Japan has also noted that similar to influenza viruses, SARS-CoV-2 may also experience a surge in prevalence during the summer months [28]. This suggests that despite being a recently emerged virus, SARS-CoV-2 exhibits a degree of seasonality akin to other viruses that cause upper respiratory tract infections [28]. However, some scholars argue that the epidemiology of SARS-CoV-2 is unique and may manifest as “wavelet” rather than seasonal surges, characterized by frequent but less severe outbreaks [29,30].

Moreover, wastewater surveillance has emerged as a crucial tool in monitoring the spatiotemporal genome diversity of SARS-CoV-2. This method has allowed researchers to track the successive appearance and disappearance of SARS-CoV-2 lineages, providing valuable data on the virus’s mutation profile and evolutionary rate. Such surveillance is vital for understanding the spread of variants and informing public health strategies [31]. These studies collectively underscore the importance of continuous genomic surveillance and epidemiological analysis in managing the ongoing challenges posed by SARS-CoV-2 and its variants.

### 3.3. Coinfection with SARS-CoV-2 and Influenza Virus

Coinfection with SARS-CoV-2 and influenza virus presents unique epidemiological challenges, as both viruses share similar transmission routes and can exacerbate disease severity when occurring simultaneously. The prevalence and impact of such coinfections have been a subject of study across various regions and populations.

As previously mentioned, the epidemic peaks of SARS-CoV-2 and influenza viruses occur in the winter and spring seasons, and they may also show a surge in the summer, indicating that SARS-CoV-2 shares a similar epidemic trend with influenza viruses [28]. Although not coinciding simultaneously with the peak of influenza A [32], the peak of COVID-19 coincides with the influenza season. This not only increases the misdiagnosis rate and places an additional burden on the healthcare systems [33], but also inevitably leads to coinfections due to their cocirculation [34]. For instance, when COVID-19 first emerged, it coincided with the high activity of seasonal influenza in China, with over 40% of the confirmed COVID-19 cases testing positive for serological IgM against influenza A virus, while the seropositive rate for influenza B virus was 7.5% [35,36]. Around the same time in Iran, 22% of the deceased COVID-19 patients tested positive for influenza infection [37,38].

Although SARS-CoV-2 and influenza viruses often coinfect with other respiratory viruses, a recent meta-analysis found that IAV is the most common virus coinfecting with SARS-CoV-2 [38]. Compared with individuals infected with a single virus, those who have both COVID-19 and influenza tend to experience more severe symptoms and outcomes [39,40]. Research has indicated that patients with coinfections face a significantly greater likelihood of hospitalization (85.7% versus 6.7%) and are at increased risk of developing complications such as acute hypoxic respiratory failure, acute respiratory distress syndrome (ARDS), cardiac injury, and acute kidney injury (AKI) than those with only SARS-CoV-2 infection [41,42].

The coinfection rates are also higher in the elderly than in adults [38,43]. As immunity declines, particularly among older adults, and with the relaxation of public health and social measures, the cocirculation and coinfection of COVID-19 and influenza during the upcoming seasonal outbreak present a new and significant health risk [44]. Therefore, it is imperative to ascertain the reasons and mechanisms behind the increased susceptibility and severity of coinfections in the elderly population.

Another unique aspect of COVID-19 and influenza viruses is that the influenza virus can enhance the infection of SARS-CoV-2. Research has indicated that following IAV infection, A549 cells, which are a type of hypotriploid alveolar basal epithelial cell that is susceptible to IAV but typically not supportive of SARS-CoV-2 infection, exhibit a two- to three-fold increase in ACE2 mRNA levels [8]. Moreover, when IAV and SARS-CoV-2 coinfection occurs, the ACE2 mRNA level in A549 cells increases by as much as 28-fold [8]. The enhancement of SARS-CoV-2 infectivity mediated by IAV is negated upon the knockout of ACE2 [8]. This evidence suggests that IAV significantly promotes the infectivity of SARS-CoV-2.

Coinfection is also associated with increased viral loads of influenza and SARS-CoV-2 in the respiratory system [8,45]. Compared with a single infection with either SARS-CoV-2 or IAV, this dual infection not only extends the duration of the initial viral infection but also leads to heightened immune cell infiltration and elevated levels of inflammatory cytokines in the bronchoalveolar lavage fluid [46]. Furthermore, coinfection leads to severe lymphopenia in peripheral blood, resulting in reduced total IgG levels, neutralizing antibody titers, and CD4^+^ T cell responses to each virus [47]. In a study conducted in Burkina Faso, the Omicron and Delta variants of SARS-CoV-2 were found to be the most prevalent, with coinfections of these variants with influenza being relatively uncommon. Continuous surveillance was recommended due to the public health implications of these coinfections [48]. Similarly, in Hamadan province, Iran, a study reported that 1.35% of the patients with COVID-19 symptoms were coinfected with both SARS-CoV-2 and influenza, highlighting the need for vigilant monitoring of respiratory infections during the pandemic [49].

The interaction between SARS-CoV-2 and influenza viruses can also influence the severity of the disease. In Egypt, coinfection with these viruses was associated with severe disease outcomes, including increased rates of pneumonia, ICU admissions, and mortality, particularly among those coinfected with influenza B and A/H3 strains [6]. This aligns with findings from a study in central Missouri, which demonstrated a high prevalence of coinfections during the 2021–2022 influenza season, with significant differences in coinfection rates between the Delta and Omicron waves of SARS-CoV-2 [50]. Moreover, the pathophysiological interactions between these viruses have been explored in experimental models. In the K18-hACE2 transgenic mouse model, coinfection with SARS-CoV-2 and influenza A virus resulted in prolonged viral shedding, increased immune cell infiltration, and elevated inflammatory cytokine levels, leading to severe pneumonia and lung damage. This study underscored the potential for coinfections to impair immune responses, including reduced neutralizing antibody titers and CD4+ T cell responses [47]. In addition to these findings, a study from the Democratic Republic of Congo reported a 13.9% prevalence of SARS-CoV-2 among patients with flu-like symptoms, with influenza A being the most common co-occurring infection. This study emphasized the importance of testing for multiple respiratory viruses to ensure accurate diagnosis and effective disease management [51].

Overall, the epidemiological features of coinfection with SARS-CoV-2 and influenza virus underscore the complexity of managing respiratory infections during the pandemic. These studies highlight the need for integrated surveillance systems and comprehensive testing strategies to better understand and mitigate the impact of such coinfections on public health. On the basis of the above description, we have summarized the comparisons between SARS-CoV-2 and the influenza virus, as well as their coinfection mechanisms, providing a new perspective for understanding the dual infection of these two viruses (Table 1).

## 4. Mechanisms That Increase the Susceptibility to and Severity of Viral Diseases in Older Adults

The elderly population exhibits significant heterogeneity, with considerable differences in self-care abilities, comorbidities of chronic diseases, nutritional status, and access to healthcare, which are not only associated with individual genetics, lifestyle, and social status but also influenced by local socioeconomic factors and social support systems [52]. However, aging, particularly inflammaging and immunosenescence, are common issues prevalent among the elderly population. Therefore, in this section, we explore the intricate relationships among aging, inflammation, and the immune system and how these factors contribute to increased morbidity and mortality among elderly individuals with infectious diseases (including diseases caused by coinfections). We delve into the concepts of inflammaging, immunosenescence, and immune system remodeling, which collectively heighten the vulnerability of older adults to infections. We also discuss the exacerbation of pre-existing inflammatory states by viral infections, the impact of immunosenescence on susceptibility to and mortality rates from viral infections, and the weakening of both innate and adaptive immune responses with age. Understanding these mechanisms is crucial for developing effective prevention and treatment strategies to protect the aging population from the devastating effects of viral infections.

### 4.1. Inflammaging and Immunosenescence Increase Morbidity and Mortality

Considering the significant increase in the risk of severe illness and death among elderly people with infectious diseases, it is crucial to delve into the underlying causes to develop effective prevention and treatment strategies. Age-related inflammation, immunosenescence, and immune system remodeling are considered the main reasons for increased susceptibility to infections, and these factors work together to increase the vulnerability of elderly individuals to pathogens [53].

#### 4.1.1. Viral Infections May Exacerbate the Pre-Existing Inflammatory State in Elderly Individuals

Inflammaging, a type of chronic inflammation associated with aging, is driven by the accumulation of senescent cells, which alter DNA methylation and chromatin structure [54,55,56]. This leads to the senescence-associated secretory phenotype (SASP), increasing the secretion of pro-inflammatory cytokines, chemokines, soluble receptors, metalloproteinases, and growth factors [57,58,59]. In the body of an older adult, inflammation results in a heightened inflammatory state [56]. Any additional inflammation related to infection could surpass the tolerable threshold, leading to severe tissue damage, disease, and even death. Notably, COVID-19 fatalities are predominantly observed in adults over the age of 65 and in those with pre-existing inflammatory conditions such as diabetes and obesity [60,61]. These findings suggest that viral infections may exacerbate an already elevated inflammatory state due to the increased burden of senescent cells associated with increasing age.

#### 4.1.2. Immunosenescence Increases Susceptibility to and Mortality Rates from Viral Infections

Immunosenescence refers to a decline in the immune system and is closely linked to inflammatory responses. Over time, the immune response to pathogens weakens, and the effectiveness of vaccines often diminishes with age [62]. The ability to trigger acute inflammation supports survival, whereas a lack of response to acute inflammation is associated with increased blood C-reactive protein (CRP) levels and an increased risk of death [63]. This decline results in heightened susceptibility to various infectious diseases, including common infections that are relatively harmless to healthy adults [56]. Immunosenescence and failed protective innate immune responses may also contribute to the high mortality rates among older adults with monoinfections and coinfections of SARS-CoV-2 and influenza viruses [34,64,65].

#### 4.1.3. Aging Weakens the Innate Immune Response, Increasing Viral Susceptibility

Aging diminishes the innate immune response, increasing the susceptibility of elderly individuals to viral infections, particularly in the context of COVID-19 and influenza coinfections. Dendritic cells (DCs), key antigen-presenting cells (APCs), activate helper T cells via MHC class II molecules (MHC-II), but their function decreases with age, characterized by reduced migration, phagocytosis, and altered cytokine production, including increased TNF-α and IL-6 and decreased IL-10, which hinders antigen presentation and the adaptive immune response [66,67,68]. Despite maintaining stable counts, neutrophils suffer from reduced phagocytosis and chemotaxis in elderly individuals, potentially exacerbating tissue damage and localized inflammation due to misdirected movement and the action of neutrophil elastases [69,70]. Natural killer (NK) cells, which bridge innate and adaptive immunity, directly eliminate infected cells, but their cytotoxic potential decreases with age, which is influenced by a decrease in IL-2 levels and responsiveness, affecting both NK cell and CD8^+^ T cell functions [56,71].

Macrophages, which are critical for initiating and resolving inflammation, face a disrupted balance with age, promoting a pro-inflammatory state and contributing to inflammation [56,72]. While their phagocytic ability persists, they can be influenced by tissue microenvironments, and aging is associated with an increase in pro-inflammatory monocyte subsets, leading to an imbalance in their functions and exacerbating inflammation [73,74]. Overall, aging impairs the functionality of immune cells, including DCs, neutrophils, NK cells, and macrophages, weakening the innate immune response and predisposing elderly individuals to more severe outcomes in viral coinfections.

#### 4.1.4. Aging Weakens the Adaptive Immune Response, Increasing Viral Susceptibility

Aging weakens the adaptive immune response, increasing susceptibility to viral infections, particularly in the context of COVID-19 and influenza coinfections. The adaptive immune system, comprising B cells, CD4^+^ T cells, and CD8^+^ T cells, is critical for fighting viral infections [75]. However, aging leads to a decrease in naive B cells, affecting the production of antibodies against new antigens and predisposing individuals to autoimmunity due to defects in the bone marrow and lymph nodes, as well as reduced Blimp-1 expression [76,77,78,79]. Age-associated B cells (ABCs) accumulate, contributing to self-reactive antibodies and autoimmune conditions [79].

T cell aging begins with thymic involution, potentially due to decreased hematopoietic stem cell progenitors or stromal niche issues, leading to a reduced production of naive T cells and a less diverse T cell receptor (TCR) repertoire [80,81,82]. This results in an increased regulatory T cell (Treg)/effector T cell (Teff) ratio and a decrease in naive Tregs with age [83]. Aging also impairs thymic medullary epithelial cells, leading to self-reactive T cells and peripheral inflammation [84]. Additionally, increased T helper 17 (Th17) activity contributes to the inflammatory state [85].

**Figure 3:**
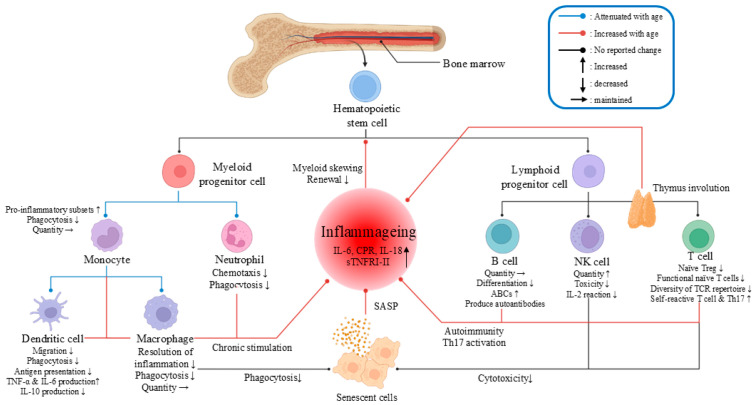
Aging, senescent cells, and inflammaging in chronic inflammation. As age increases, the accumulation of senescent cells and the associated SASP lead to a persistent low-grade inflammatory state, known as inflammaging [56,89]. This inflammatory state tends to affect the myeloid skewing of HSCs, promoting innate immune responses while weakening adaptive immunity [56]. Innate immune cells derived from myeloid progenitor cells exhibit a decline in their migratory and antigen-presenting capabilities, indicating a weakened anti-inflammatory capacity [66]. However, their ability to secrete pro-inflammatory cytokines increases with age. On the other hand, adaptive immune cells, such as T and B cells, exhibit reduced cytotoxicity [87], leading to a weakened ability to clear senescent cells. Moreover, an increase in the subset of immune cells that secrete pro-inflammatory cytokines also contributes to elevated inflammation levels in elderly individuals [73]. The accumulation of senescent cells enhances the pro-inflammatory capacity of the immune system in elderly individuals while reducing its anti-inflammatory function, directly contributing to inflammation. Concurrently, the immune system’s diminished ability to clear senescent cells indirectly promotes inflammaging through the SASP, creating a positive feedback loop. SASP: senescence-associated secretory phenotype; HSCs: hematopoietic stem cells.

Compared with those of younger cells, the size and clonality of CD8^+^ T cell compartments decrease during aging, and their cytotoxicity is reduced [86,87]. Memory CD8^+^ T cells, despite increasing in number, exhibit cell cycle arrest and expression of the senescence marker KLRG1, indicating functional loss [88]. Immunosenescence, which is intertwined with inflammaging, results from complex interactions within the immune system and the SASP from senescent cells, exacerbating inflammation (Figure 3) [56]. Aging immune cells enhance autoimmune responses and pro-inflammatory Th17 activity and impair immune surveillance, whereas innate immune cells drive inflammation through myeloid skewing. Senescent macrophages may also fail to clear senescent cells, further exacerbating inflammation [56].

In summary, aging diminishes the adaptive immune response, increasing vulnerability to viral coinfections such as COVID-19 and influenza by impairing B and T cell functions and contributing to a chronic inflammatory state.

### 4.2. Aging Leads to Increased Viral Load in Older Patients

Immunosenescence suppresses the generation of protective adaptive immunity mediated by B cells and T cells; influences the phenotype, function, and receptor repertoire of B cells and T cells in elderly individuals; and hinders the protective immune response [56]. This condition may increase the viral load [90,91], thereby increasing the susceptibility to and severity of disease in older adults. Furthermore, numerous studies have indicated that aging can also increase the viral load by influencing metabolism and increasing the number of virus-specific receptors.

In summary, aging either directly or indirectly elevates the viral load in elderly individuals, thereby posing a significant threat to their lives.

#### 4.2.1. Aging Contributes to Obesity, Which Increases the Viral Load

A cross-sectional, real-world study of 15.8 million adults identified divergences in the prevalence of overweight and obesity by age within each sex and revealed that the prevalence of both overweight and obesity peaked in middle-aged individuals (55–59 years old) in males and in older individuals (65–69 years old) in females [92]. Obesity is considered one of the risk factors for influenza and COVID-19 [93]. Epidemiological studies have shown that obesity increases the risk of severe complications and death after influenza virus and SARS-CoV-2 infection, especially among elderly individuals [93,94,95].

A study from Maryland, USA, quantified viral RNA in participants infected with respiratory viruses and reported a positive correlation with body mass index (BMI), identifying obesity as a key factor in prolonging the virus shedding time and increasing the viral load in adults [96]. Obese individuals are in a state of chronic inflammation, which inhibits both innate and adaptive immune responses [97], leading to a weakened immune response to viruses, bacteria, and parasites, including the influenza virus and SARS-CoV-2 [98,99], thereby increasing the viral load and the susceptibility to and severity of diseases in obese individuals.

In summary, metabolic changes in elderly individuals increase their likelihood of becoming obese, which in turn is a significant factor in the onset and progression of severe diseases.

#### 4.2.2. Aging Increases the Gene Expression of Virus-Specific Receptors

As previously discussed, both SARS-CoV-2 and influenza viruses bind to host cell membranes, with SARS-CoV-2 utilizing its S protein in conjunction with ACE2 [100]. Research has shown that higher ACE2 gene expression levels increase the ability of the virus to invade and proliferate within cells, thereby increasing disease susceptibility [101]. Notably, ACE2 expression levels increase with age [102], facilitating viral entry in older adults and potentially leading to higher viral loads and more severe disease outcomes.

Similarly, in obesity, ACE2 expression is elevated [103], suggesting that aging can indirectly increase ACE2 gene expression, thereby increasing the viral load. Studies on SARS have shown that cholesterol removal from host cells inhibits virus release and replication, while the addition of cholesterol and lipids can counteract this inhibition, highlighting the role of lipids in viral replication [104]. It is hypothesized that SARS-CoV-2 can infect adipocytes via ACE2, with those high in triglycerides and cholesterol potentially accelerating viral propagation and worsening infection.

In the context of influenza, the virus also exploits host cell receptors, although the specific mechanisms may differ. However, the principle that an aged or compromised metabolic state can increase viral susceptibility remains relevant. The increased ACE2 expression in older individuals and those with metabolic disorders such as obesity can, thus, be seen as a common factor exacerbating the impact of both SARS-CoV-2 and influenza virus infections, leading to increased viral loads and increased severity of illness. In essence, the interplay between ACE2 expression, aging, and metabolic health is crucial in understanding the increased vulnerability of older and obese populations to viral infections.

### 4.3. Aging Increases Severity Due to Frailty and Chronic Diseases

Aging enhances the adverse clinical outcomes of viral infections, such as COVID-19 and influenza, due to increased frailty and the prevalence of chronic diseases among elderly individuals [2,105]. As individuals age, immunosenescence impairs the body’s ability to mount an effective defense against new pathogens. This decline in immune function is exacerbated by chronic conditions such as diabetes, cardiovascular diseases, and respiratory illnesses, which are more common in older adults and can further suppress the immune response [2,106,107]. The combination of immunosenescence, frailty, and chronic diseases not only increases the risk of infection but also leads to more severe outcomes, including higher rates of hospitalization and mortality. Notably, older adults and patients with multiple comorbidities are more susceptible to coinfections [107,108], likely because of their relatively weaker immune defenses against respiratory viruses, as mentioned earlier. This susceptibility may lead to an increased risk of mortality in these groups, a point supported by animal experiments and case statistical analyses. Compared with single viral infections, mice and ferrets coinfected with both viruses presented more severe pulmonary and lung damage [46,47]. Studies in humans have also revealed that coinfection can lead to more severe clinical outcomes, including AKI, acute heart failure, secondary bacterial infections, and intensive care unit (ICU) admissions [7]. Aging, with its associated frailty and chronic diseases, significantly amplifies the vulnerability of elderly individuals to viral infections, particularly coinfections with COVID-19 and influenza, resulting in severe clinical outcomes and increased mortality. This underscores the importance of focused preventative strategies and tailored medical interventions for this population.

## 5. Potential Clinical Manifestations and Complications of SARS-CoV-2 and Influenza Coinfection in Elderly Individuals

**Figure 4:**
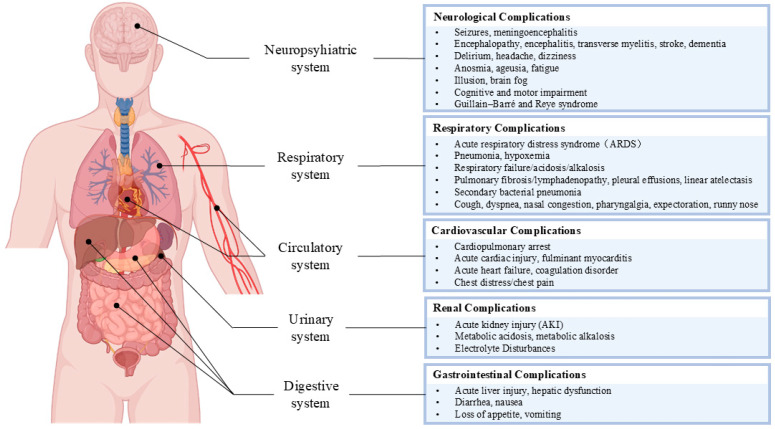
Clinical manifestations and complications of coinfection with influenza and COVID-19 in the elderly. The common symptoms associated with SARS-CoV-2 and influenza coinfection, as analyzed in a large number of cases, include fever, cough, dyspnea, nasal congestion, pharyngalgia, myalgia, fatigue, headache, and expectoration [36,40,109]. Moreover, in high-risk populations such as elderly individuals, coinfection with SARS-CoV-2 and influenza can lead to not only respiratory complications but also other severe adverse outcomes. The most common complications prior to death include ARDS, AKI, acute cardiac injury, and liver dysfunction [110].

This section delves into the clinical manifestations of SARS-CoV-2 and influenza coinfections across various organ systems (Figure 4 and Table 2). We explored how these viral coinfections can lead to severe complications in the neuropsychiatric, respiratory, cardiovascular, urinary, and digestive systems. Understanding these manifestations is crucial for medical professionals, as it aids in the early identification and management of these critical conditions, particularly in vulnerable populations such as elderly individuals and those with chronic comorbidities. The following discussion highlights the interplay between viral infections and the body’s physiological responses, leading to multifaceted clinical outcomes that demand comprehensive care.

### 5.1. Viral Sepsis Involves Multiorgan Dysfunction

Severe or critical COVID-19 patients may present with shock symptoms such as cold extremities and weak pulses without hypotension, meeting the Sepsis-3 criteria for sepsis and septic shock [139]. Most bacterial cultures are negative, suggesting that SARS-CoV-2 is the sole cause [140], leading to the term “viral sepsis” [137]. This also applies to influenza coinfections, which can cause more severe symptoms [137,138]. Older adults are at greater risk for worse outcomes [141,142].

**Figure 5:**
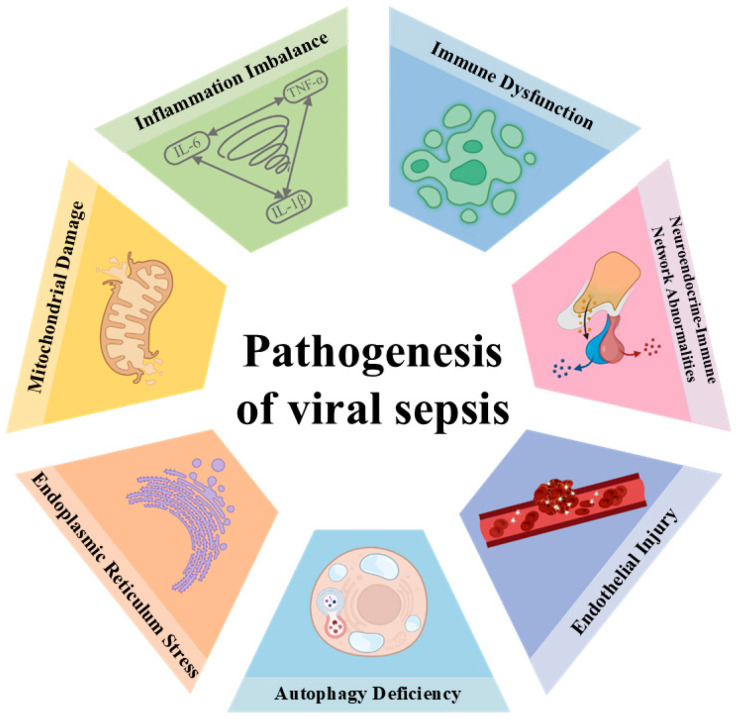
Pathogenesis of sepsis in elderly patients with SARS-CoV-2 or influenza. The pathogenesis of sepsis primarily involves an imbalance in inflammation, immune dysfunction, mitochondrial damage, neuroendocrine–immune network dysregulation, endoplasmic reticulum stress, endothelial injury, and autophagy deficiency [138,143]. These factors interact and contribute to each other, and with increasing age, they tend to be in an imbalanced state, which is one of the important reasons why elderly individuals are more prone to developing severe and critical conditions after being infected with SARS-CoV-2 or influenza virus.

Viral sepsis involves inflammatory imbalances, immune dysfunction, mitochondrial damage, neuroendocrine–immune disruptions, endoplasmic reticulum stress, endothelial injury, and impaired autophagy, leading to multiorgan dysfunction and septic shock (Figure 5) [138,143]. Inflammatory imbalance triggers a cytokine storm, especially in older adults, leading to complications such as septic shock [143,144]. Coinfections may increase neutrophil activation, contributing to cytokine storms [110]. Survivors face postsepsis immunosuppression with compensatory anti-inflammatory response syndrome (CARS), which affects the clearance of infections and reactivation of viruses [143].

**Figure 6:**
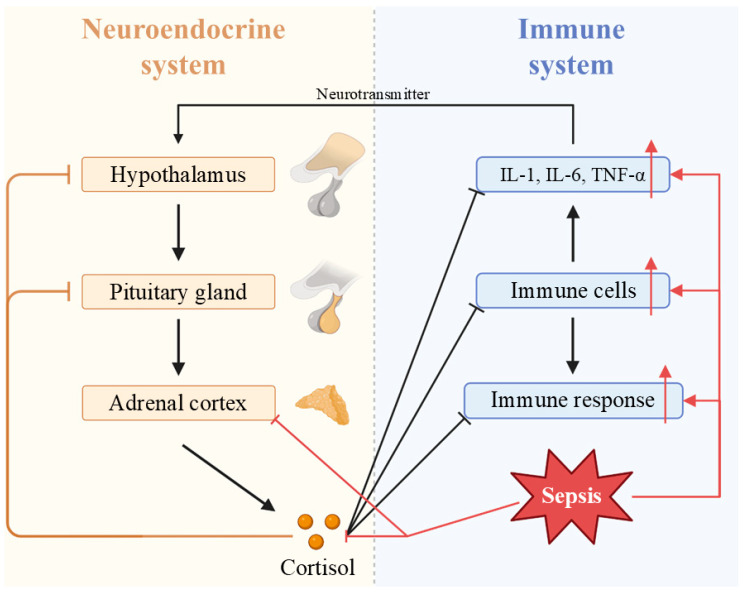
HPA axis and immune response regulation in sepsis: balancing inflammation and immunosuppression. The HPA axis modulates the immune response through the neuroendocrine pathway, releasing cortisol to maintain immune balance [154]. Concurrently, inflammatory mediators produced by immune activation can regulate the neuroendocrine system via feedback, ensuring that the immune response effectively eliminates pathogens without triggering excessive inflammation. However, in the context of sepsis, the function of the HPA axis may be compromised, along with a relative insufficiency of adrenal cortical hormones, potentially leading to systemic inflammation and immunosuppression, thereby impacting the clinical prognosis of patients. IL-1: interleukin-1; IL-6: interleukin-6; TNF-α: tumor necrosis factor-alpha.

**Figure 7:**
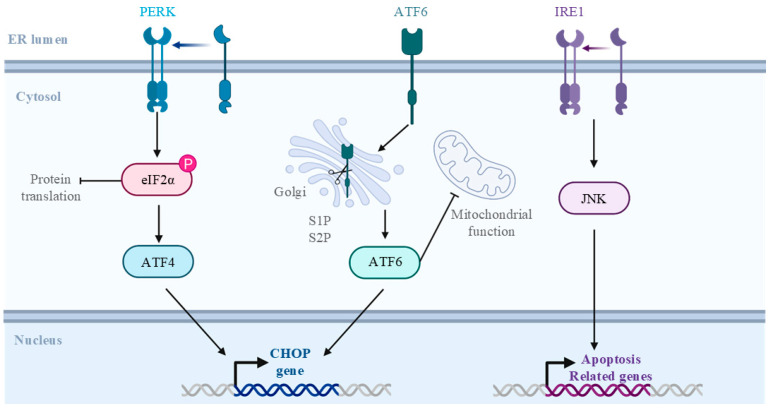
Protein kinases PERK, ATF6, and IRE1 in the ER stress response and apoptosis. PERK is a protein kinase located on the ER membrane. When proteins are properly folded, PERK forms a stable complex with molecular chaperones such as BIP/GRP78. Misfolded proteins bind to BIP/GRP78, competing with PERK for interaction, which leads to the release and activation of PERK. Activated PERK undergoes oligomerization and autophosphorylation, which then phosphorylates eIF2α. Phosphorylated eIF2α inhibits protein translation and synthesis, reducing the load on protein folding in the ER and providing a protective effect for the cell. As the duration and intensity of the stress response increase, phosphorylated eIF2α induces the transcriptional activation of the transcription factor ATF4, which in turn promotes the expression of the apoptotic signaling molecule CHOP [148,155,156]. ATF6 is a type II transmembrane protein located on the ER membrane. During ERS, ATF6 is transported to the Golgi and is cleaved and activated by S1P and S2P. This protein subsequently migrates to the nucleus under the guidance of nuclear localization signals, where it induces the expression of CHOP and other proapoptotic signaling molecules [148,155,156]. The activation of ATF6 may also affect the function of the mitochondria by influencing the exchange of calcium ions between the ER and the mitochondria, thereby exacerbating mitochondrial damage [157]. Like PERK, IRE1 is also a protein kinase located on the ER membrane, and its activation mechanism mirrors that of PERK. Under conditions of ERS, activated IRE1 can recruit and activate JNK, which then phosphorylates and inhibits the activity of antiapoptotic proteins in the Bcl-2 family, thereby promoting apoptosis [148,155,156]. On the other hand, activated IRE1 also recruits the cytoplasmic regulatory protein TRAF-2, which in turn activates Caspase-12, initiating the caspase cascade and mediating apoptosis [158]. ER: endoplasmic reticulum; PERK: protein kinase R-like endoplasmic reticulum kinase; eIF2α: eukaryotic initiation factor 2 alpha; ATF-4: activating transcription factor 4; ATF-6: activating transcription factor 6; CHOP: C/EBP homologous protein; S1P: serine protease site-1; S2P: serine protease site-2; IRE1: inositol-requiring enzyme 1; JNK: c-Jun N-terminal kinase.

Hypoxia and inflammation during viral sepsis increase free radicals, disrupting the mitochondrial electron transport chain (ETC) and leading to oxidative stress and apoptosis, potentially causing multiorgan failure and death [145,146]. Viral sepsis impacts the hypothalamic–pituitary–adrenal (HPA) axis, causing adrenal insufficiency and affecting patient prognosis (Figure 6) [147]. ERS results from the accumulation of unfolded proteins due to UPR dysfunction (Figure 7) [148]. Endothelial cells express tissue factor (TF) and adhesion molecules, promoting thrombus formation, and injury impairs anticoagulant mechanisms, exacerbating coagulopathy and increasing the severity of viral sepsis [149,150]. Autophagy deficiency with age may worsen viral sepsis progression [151], and studies have shown that autophagy suppression intensifies sepsis and organ damage [152,153].

In summary, viral sepsis in COVID-19 and influenza coinfections is characterized by a complex interplay of inflammatory, immunological, and cellular responses that can lead to severe clinical outcomes, particularly in older adults.

### 5.2. Clinical Manifestations of Viral Coinfection in the Neuropsychiatric System

The findings in COVID-19 patients and influenza patients, particularly older adults, suggest a correlation between age and neurological issues. Coinfections with these viruses can lead to various neurological manifestations, including delirium, headache, dizziness, fatigue, anosmia, and ageusia [109,111,115,116], with complications such as seizures and meningoencephalitis being frequent [112]. Other conditions, such as encephalopathy, encephalitis, transverse myelitis, stroke, dementia, illusion, and brain fog, have also been observed [4,113,114]. Cognitive and motor impairments can result in long-term sequelae such as Parkinson’s disease and Alzheimer’s disease [117], whereas Guillain–Barré syndrome and Reye syndrome are less common complications [119,120].

**Figure 8:**
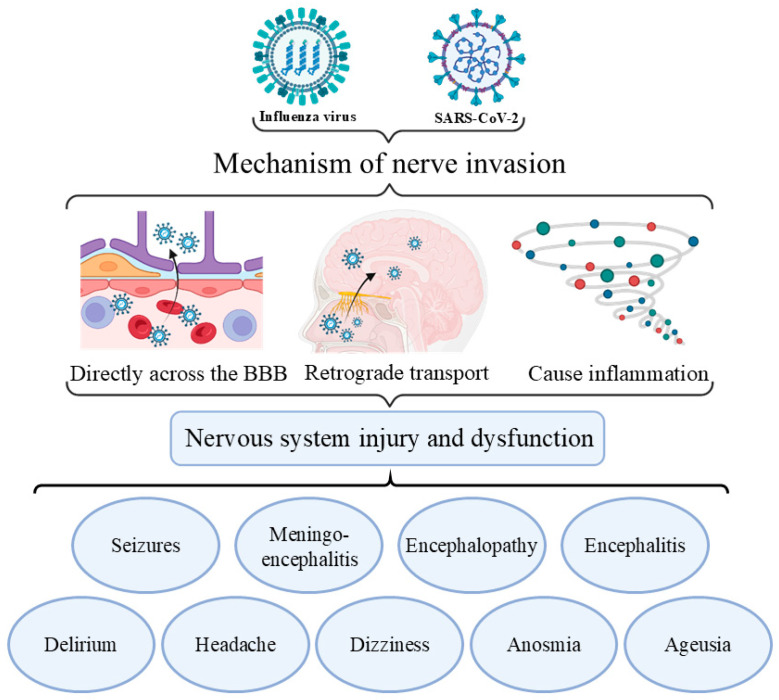
Neurological complications caused by SARS-CoV-2 and influenza virus. SARS-CoV-2 and influenza virus can cross the BBB through various means, either directly or indirectly, causing neurological damage or dysfunction, which can subsequently lead to a range of neurological complications and clinical manifestations.

Influenza viruses and SARS-CoV-2 invade the central nervous system (CNS) primarily through the direct invasion of the blood–brain barrier (BBB), retrograde transport through olfactory neurons and the vagus nerve, or the induction of inflammatory responses that compromise the BBB (Figure 8) [159,160,161,162,163,164,165,166]. The detection of viral RNA in the cerebrospinal fluid confirms the neurotropic properties of these viruses [159]. They can trigger inflammation imbalances, potentially leading to cytokine storms, encephalitis, and systemic inflammation. This inflammation can cause endothelial dysfunction, compromising the BBB and allowing inflammatory mediators to access the brain, exacerbating neurological damage [167]. The injured endothelium may also activate the coagulation system, leading to thrombotic disorders such as ischemic stroke [168]. Systemic inflammation can induce multiorgan failure, indirectly mediating brain damage.

In summary, COVID-19 and influenza cause neurological symptoms and complications more frequently in severe cases and in older adults, warranting concern. Common symptoms in coinfections include delirium, headache, dizziness, fatigue, anosmia, and ageusia [109,111,115,116], with seizures and meningoencephalitis being frequent complications [112], along with other manifestations [4,113,114]. SARS-CoV-2 and influenza viruses can penetrate the BBB and attack the brain through various means, and they can also indirectly affect brain function through cytokine storms.

### 5.3. Clinical Manifestations of Viral Coinfection in the Respiratory System

Coinfections with SARS-CoV-2 and influenza typically present with respiratory symptoms such as cough, dyspnea, and nasal congestion, which are often self-limiting [109]. However, severe complications such as pneumonia, ARDS, and linear atelectasis are common and affect both respiratory and systemic health [40]. Viral pneumonia, often caused by influenza A and B and coronaviruses, is characterized by interstitial pneumonia and mononuclear cell infiltration in alveolar septa, leading to alveolar edema and gas exchange [169]. This can result in respiratory failure and hypoxemia, potentially leading to acidosis [107].

Inflammatory mediators cause bronchospasm, reduce lung volume and compliance, and disrupt the ventilation–perfusion ratio, which can lead to respiratory failure and respiratory acidosis [107]. A cytokine storm, indicated by increased pro-inflammatory cytokines, is associated with severe COVID-19 and can cause ARDS and extensive pulmonary pathology, including linear atelectasis and pulmonary fibrosis [36,170].

SARS-CoV-2 overactivates the renin–angiotensin system (RAS) system by decreasing ACE2 expression, leading to increased angiotensin II (Ang II) levels and lung injury [171]. This disruption activates interferon responses, promotes inflammatory bodies, induces free radicals, and triggers oxidative stress, exacerbating lung injury in severe COVID-19 [172].

In summary, coinfections with SARS-CoV-2 and influenza can lead to severe respiratory complications due to cytokine storms, impaired gas exchange, and systemic inflammation, emphasizing the need for close surveillance and management of these high-risk patients [110].

### 5.4. Clinical Manifestations of Viral Coinfection in the Cardiovascular System

A retrospective study of 285 COVID-19 patients, including 36 with coinfections of COVID-19 and influenza, revealed that elderly patients with coinfections are at increased risk of adverse clinical events such as acute heart failure, intensive care unit (ICU) admission, and fatal complications in other systems [7]. Patients with pre-existing cardiovascular comorbidities are particularly at risk of developing cardiac complications [173].

Cardiovascular complications in patients with SARS-CoV-2 and influenza coinfection include cardiopulmonary arrest, acute cardiac injury, fulminant myocarditis, acute heart failure, coagulation disorders, and chest distress/pain [40,112,128]. Laboratory tests have shown significant increases in the levels of markers of cardiac injury and heart failure, such as creatine kinase (CK), creatine kinase isoenzyme MB (CK-MB), cardiac troponin I (cTnI), and brain natriuretic peptide (BNP) [174]. Coagulation parameter alterations, including decreased platelet count and increases in D-dimer, fibrinogen, prothrombin time (PT), and activated partial thromboplastin time (APTT) may also occur [110,129].

Cardiovascular complications can result from the direct viral infection of the heart muscle, leading to myocardial damage and necrosis, potentially causing viral myocarditis and cardiac injury [175,176]. Inflammatory imbalances can also contribute to myocardial injury by increasing endothelial damage and thrombus formation, leading to acute coronary syndrome (ACS) [177,178]. Additionally, inflammation-induced fever, dehydration, tachycardia, increased adrenaline secretion, and hypoxemia can promote myocardial ischemia [177,179]. Acute infections can trigger coronary artery constriction, leading to ACS [177].

Influenza virus infection can trigger low-grade intestinal inflammation, altering the gut microbiota and facilitating microbial metabolite translocation, exacerbating cardiovascular damage [175]. The influenza virus can also modulate matrix metalloproteinase-13 (MMP-13) expression, contributing to arterial plaque instability [180]. SARS-CoV-2 can impair the vascular endothelial barrier, initiate cardiovascular inflammation, downregulate ACE2 expression, affecting multiple organs and triggering the release of von Willebrand factor (vWF) multimers, leading to microthrombi and multiorgan ischemia [181]. In summary, compared with single infections, coinfections with COVID-19 and influenza are associated with a greater risk of cardiovascular complications, including cardiac injury and acute heart failure [7,110]. Laboratory indicators such as D-dimer and APTT are also elevated in coinfected patients, reflecting the involvement of cytokine storms and coagulation disorders in these complications [110,129].

### 5.5. Clinical Manifestations of Viral Coinfection in the Urinary System

Research data show that SARS-CoV-2 and influenza coinfection increases the risk of kidney impairment, such as proteinuria, hematuria, anuria, or AKI, especially in elderly patients with chronic conditions such as kidney disease and diabetes. This increases the likelihood of adverse outcomes, prolonged hospitalization, and death [7,110,131,182]. Metabolic acidosis is believed to result from impaired ammonia excretion and reduced reabsorption of bicarbonate by the renal tubules [132]. Metabolic alkalosis and electrolyte disturbances (including hypokalemia and hypocalcemia) can also manifest as complications in patients with influenza and COVID-19 [133,134].

Various factors may contribute to the development of AKI during SARS-CoV-2 and influenza infection. Direct viral cytopathic effects and disruptions in renal hemodynamics are potential causes of AKI in patients [183,184]. In addition to the virus’s direct assault on renal cells, other contributing factors include the systemic hyper-inflammatory response, known as the cytokine storm; hypoxia-induced tissue damage; nephrotoxicity from medications used in treatment; and secondary infections by a range of pathogens, including other viruses, bacteria, and fungi [185]. Moreover, the pathways associated with sepsis are likely mechanisms underlying the kidney injury observed in these cases [143].

The overactivation of the RAS caused by SARS-CoV-2 (mentioned above) not only leads to acute tubular necrosis through inflammatory responses and oxidative stress but can also result in hyperaldosteronism [134]. This condition increases the loss of hydrogen ions, calcium ions, potassium ions, and other electrolytes from renal tubular cells, causing complications such as metabolic alkalosis, hypocalcemia, and hypokalemia [133,134]. In summary, coinfection with COVID-19 and influenza increases the risk of kidney impairment, particularly in elderly patients with chronic conditions such as kidney disease and diabetes [131,186]. This can lead to proteinuria, hematuria, anuria, or AKI and increase the risk of adverse outcomes, extended hospital stays, and mortality [7,110,131,182]. The primary mechanisms underlying this process include cytokine storms, hypoxia, overactivation of the RAS, and electrolyte imbalances.

### 5.6. Clinical Manifestations of Viral Coinfection in the Digestive System

Patients with SARS-CoV-2 and influenza coinfections commonly present digestive system symptoms such as diarrhea, nausea, loss of appetite, and vomiting [115,136]. Acute liver injury and hepatic dysfunction are also frequent complications in severe cases [40,135]. Abnormal liver function parameters, including elevated levels of alanine aminotransferase (ALT), aspartate aminotransferase (AST), gamma glutamyl transferase (GGT), and lactate dehydrogenase (LDH), are often observed and correlate with disease severity [110,187].

The mechanisms of liver damage in coinfections involve direct viral invasion, with SARS-CoV-2 binding to ACE2 and influenza viruses detected in damaged liver tissues [188,189]. Medications such as antipyretics and antiviral drugs can cause liver toxicity [190]. Secondary liver damage can result from immune responses and systemic inflammation, with cytokines contributing to immune liver damage [191]. Hypoxia resulting from severe viral pneumonia also affects liver detoxification, causing liver injury [192]. In summary, coinfections with SARS-CoV-2 and influenza are associated with digestive symptoms and liver complications, including abnormal liver function tests and acute liver injury, which are linked to direct viral effects, medication toxicity, immune responses, and hypoxia.

## 6. Prevention, Diagnosis, and Treatment Recommendations for SARS-CoV-2 and Influenza Coinfections

As previously highlighted, COVID-19 and influenza coinfection are linked to an increased likelihood of adverse clinical outcomes, especially among elderly individuals, which could significantly affect the management and treatment of the disease [6,7,108]. However, sharing the same transmission pathway, SARS-CoV-2 and influenza may cause a copandemic in the population, making the occurrence of coinfection inevitable [38]. Although the proportion of reported coinfection cases is currently low, some research suggests that COVID-19 and influenza coinfections may be potentially underestimated [130]. Therefore, in the context of an aging population, placing significant emphasis on the prevention, diagnosis, and treatment of COVID-19 and influenza coinfections is crucial. In this section, we have summarized the prevailing approaches to the prevention, diagnosis, and treatment of COVID-19 and influenza coinfections, hoping to provide some recommendations for clinical practice.

### 6.1. Prevention

Nonpharmaceutical interventions (NPIs) during the early period of the COVID-19 pandemic were effective in controlling the spread of influenza [38]. Studies indicate that compared with that in the prepandemic period, the positive detection rate of influenza viruses has decreased by more than 95% during the acute phase of the pandemic [193]. Following the postponement of the pandemic, which commenced after the WHO declared the end of the global emergency status of COVID-19 in May 2023, the global prevalence of seasonal influenza returned to patterns similar to those observed prior to the pandemic [193]. This situation suggests that continuing to implement some NPIs, such as maintaining social distance and wearing masks under necessary conditions, is an effective method of prevention [194], especially for the elderly, who tend to have relatively weaker immune systems. Vaccination also serves as an effective measure to prevent the occurrence and progression of severe cases of COVID-19 and influenza coinfections. Studies indicate that administering both COVID-19 and influenza vaccines concurrently can reduce the severity of coinfections, particularly among the elderly population [195]. Notably, influenza vaccination also offers a degree of protection for patients with COVID-19. Some research has shown that influenza vaccination can mitigate cardiovascular morbidity and mortality in individuals with COVID-19 [196]. Wilcox et al. reported a significant association between influenza vaccination and a reduced risk of hospitalization or all-cause mortality due to COVID-19, with an overall 24% decrease in all-cause mortality [197]. However, research suggests that the willingness to receive influenza vaccination is lower among elderly individuals and those with chronic conditions [198]. Therefore, it is essential to promote vaccination among these specific groups.

Research indicates that effectiveness for both COVID-19 and influenza vaccines experiences small but statistically significant decreases over time [199,200]. Consequently, despite being vaccinated, individuals may still be at risk of infection. While vaccinated individuals generally tend to have milder cases compared to those who are unvaccinated [197], this risk is not negligible for the elderly or those with pre-existing health conditions, highlighting the necessity of personal protective measures which was mentioned above. Given the decline in vaccine effectiveness over time [199,200], and considering that COVID-19 and influenza viruses can peak in both summer and winter-spring seasons [28], it may be beneficial to receive a vaccination approximately every six months prior to the anticipated onset of an epidemic peak.

In view of the abovementioned facts, in recent years, combination vaccines have gradually become a research focus. By consolidating multiple antigens into a single dose, combination vaccines offer several societal benefits: (1) they minimize the need for multiple injections and reduce side effects; (2) they decrease the frequency of medical visits; (3) they simplify vaccine management and storage; (4) they lower the risk of necessary injuries for caregivers; and (5) they enhance the timeliness and coverage of vaccination [201,202]. Currently, the platforms for developing combination vaccines combating SARS-CoV-2 and influenza primarily include mRNA vaccines, subunit vaccines, and viral vector vaccines. Subunit vaccines include recombinant protein and virus-like-particle (VLP) vaccines, whereas viral vector vaccines include influenza-based, VSV-based, and adenovirus vector vaccines (Table 3). These combination vaccines have generally been effective in stimulating the production of sufficient virus-specific antibodies and cellular immunity in their respective animal studies, effectively protecting experimental animals from SARS-CoV-2 and influenza virus infections [201,203,204,205,206,207,208,209,210,211,212,213,214]. Moreover, some combination vaccines also provide complete protection against coinfection with SARS-CoV-2 and influenza [203].

### 6.2. Diagnosis

In terms of diagnosis, as previously highlighted, numerous studies have shown that COVID-19 and influenza can present with similar clinical symptoms in cases of monoinfection and coinfection [7,215], making it challenging to differentiate between them based solely on clinical manifestations [216]. Moreover, due to the similar epidemic trends shared by SARS-CoV-2 and influenza, it is also difficult to distinguish among the above situations based on medical history [28]. In terms of laboratory tests, inflammatory markers (CRP, procalcitonin, and LDH) are significantly higher in cases of coinfection, but lymphocyte counts are significantly reduced [41]. But, some literature also indicates that lymphocyte counts are significantly higher in COVID-19 and influenza coinfections, and their APTT is significantly higher than in monoinfections [43]. However, most studies suggest that there are no laboratory differences between monoinfection and coinfection in terms of WBC counts, CPR, and IL-6 [217]. In terms of imaging examinations, chest radiologic findings in patients with COVID-19 and influenza coinfections show infiltrates, mild vascular congestion, and patchy diffuse bilateral infiltrates, while chest computed tomography (CT) findings are essentially consistent with those only infected by SARS-CoV-2, showing bilateral multifocal basal opacification, peripheral distribution, and mild pleural effusion [43]. This also indicates that it is challenging to differentiate between monoinfection and coinfection of COVID-19 and influenza through laboratory and imaging examinations.

In real-world clinical practice, the surge of COVID-19 cases may cause some clinicians to overlook testing for influenza [218]. Similarly, in instances where influenza is diagnosed, the potential for SARS-CoV-2 coinfection might be disregarded [218]. Furthermore, it is well established that a variety of pathogens, encompassing bacteria, viruses, fungi, mycoplasma, and chlamydia, can cause respiratory and multiorgan systemic symptoms, whether through single or coinfections. Although pathogen isolation and culture can distinguish the aforementioned pathogens, it takes too much time and may potentially fail; therefore, molecular diagnostics [7], especially multiplex genome detection, serological detection, and antigen detection assays are highly recommended. These approaches can ensure timely and appropriate treatment, leading to better outcomes. Due to the lower sensitivity of antigen detection compared to nucleic acid testing, a negative result does not necessarily rule out SARS-CoV-2 or influenza virus infection, and should be confirmed with nucleic acid testing or repeated antigen testing after 48 h. For critically ill patients with suspected COVID-19 or influenza, or both, including those with negative upper respiratory specimens, nucleic acid testing for SARS-CoV-2 and influenza virus detection can be performed on lower respiratory specimens (https://www.cdc.gov). Additionally, research suggests that the cycle threshold (Ct) values from PCR tests conducted on nasopharyngeal swabs during the early stages of coinfection may offer valuable insights into the subsequent progression of the disease [7].

### 6.3. Treatment

In the treatment of COVID-19 and influenza coinfections, notable heterogeneity exists among the therapeutic strategies proposed by various studies. A systematic review, which synthesized findings from numerous studies, indicated that in the treatment of coinfections of COVID-19 and influenza, the most commonly provided form of support is oxygen/ventilation [40]. Furthermore, research has identified arbidol, oseltamivir, and vasopressors as the most frequently used pharmaceutical treatments in SARS-CoV-2 and influenza coinfections, highlighting their significant roles in therapeutic strategies [40].

In the treatment of COVID-19 and influenza coinfection, the use of antiviral drugs is crucial. Arbidol, a broad-spectrum antiviral drug, is capable of treating a variety of viral infections, including COVID-19 and influenza viruses. And, as for IAV and IBV, oseltamivir (NA inhibitor, taken orally, usually twice daily for 5 days, suitable for children aged 14 days and older and adults); Zanamivir (NA inhibitor, an inhaled agent, usually twice daily for 5 days, suitable for children aged 7 years and older and adults, not recommended for patients with respiratory diseases); Peramivir (NA inhibitor, administered intravenously as a single dose, suitable for children aged 6 months and older); and baloxavir (a cap-dependent endonuclease inhibitor, single oral dose, suitable for children aged 5 years and older without chronic conditions and people aged 12 years and older) can be chosen (https://www.cdc.gov). Studies show that influenza antiviral drugs are most effective when started within two days of illness onset (https://www.cdc.gov). Amantadine and Rimantadine are primarily used to treat IAV, but their use gradually decreases due to resistance issues.

For SARS-CoV-2, Paxlovid (contains nirmatrelvir and ritonavir, taken orally twice daily for 5 days, should be started as soon as possible within 5 days of symptom onset, and is suitable for children aged 12 years and older who weigh at least 40 Kg and adults); remdesivir (an inhibitor of the SARS-CoV-2 RNA-dependent RNA polymerase, suitable for children aged 28 days and older who weigh at least 3 Kg and adults, a 3-day course of intravenous remdesivir initiated within 7 days of symptom onset is the second preferred treatment option after ritonavir-boosted nirmatrelvir); and molnupiravir (a broad-spectrum drug against RNA viruses, recommended for use if the above medications cannot be used or are unavailable, taken orally twice daily for 5 days, and should be started as soon as possible within 5 days of symptom onset, suitable for adults, contraindicated in pregnant women) can be chosen (https://www.cdc.gov). Additionally, 17 approved novel candidate drugs for COVID-19 treatment were identified by mapping the gene networks associated with the pathogenicity of SARS-CoV-2 [219], and baloxavir also showed inhibitory activity against SARS-CoV-2 [220]. The in vitro study also indicated that in comparison with monotherapy using molnupiravir or baloxavir, a combination therapy of molnupiravir and baloxavir may be more advantageous for the early treatment of patients coinfected with SARS-CoV-2 and influenza [220].

Because coinfections are more susceptible to secondary bacterial infections than single infections are, the use of antibiotics such as azithromycin, ceftriaxone, vancomycin, amoxicillin, and doxycycline is also frequently employed in the treatment of patients with coinfections in order to prevent secondary bacterial infections [7,40]. For elderly patients with chronic comorbidities such as cardiovascular diseases, macrolides should be cautiously selected because of their potential to increase cardiovascular mortality risk [221]. Some studies suggest that in cases of severe pulmonary complications due to coinfection, steroids should be used to prevent and treat respiratory distress. However, the use of steroids should be approached with caution, as they may have adverse effects on the incidence and mortality rates of these patients [115]. Furthermore, tailored treatment strategies should be established for the intervention and management of patients with coinfections, aiming to mitigate the potential for severe outcomes [38].

### 6.4. Conclusions

In summary, coinfection with COVID-19 and influenza increases the risk of severe disease progression, especially in elderly individuals [6,7,108]. Therefore, attention to the prevention, diagnosis, and treatment of coinfections is warranted. For prevention, it is important to follow the guidelines for NPIs, which include strengthening personal protective measures and getting vaccinated [194,195]. In terms of diagnosis, given the propensity for clinicians to overlook coinfections, molecular diagnostics for hospitalized patients are recommended [218]. In terms of treatment, ventilation and antiviral drugs are most commonly used, with antibiotics also being frequently prescribed [7,40]. Steroids should be used judiciously and only in specific situations [38,115].

## 7. Conclusions and Prospects

As aging impairs the immune function of elderly individuals, they become more vulnerable to infections, notably SARS-CoV-2 and influenza. This review examines their coinfection dynamics, increased susceptibility in elderly individuals, and associated complications across major body systems. We outline strategies for prevention, diagnosis, and treatment, aiming to mitigate dual infection risks and enhance the health of older adults.

There is an urgent need for further research into the mechanisms underlying the increased susceptibility of elderly individuals to viral infections. This includes elucidating the role of immunosenescence, the impact of coinfections, and the influence of comorbidities on disease severity. Additionally, long-term follow-up of survivors of coinfections with COVID-19 and influenza can provide insights for postacute care strategies following dual infections. Socioeconomic and demographic factors, such as access to healthcare, comorbidities, nutritional status, and living conditions, can significantly influence the risk and outcomes of COVID-19 and influenza coinfections among the elderly. Therefore, investigating the differences in preventive and therapeutic strategies across various regions may provide valuable insights into the optimal practices for the diagnosis and treatment of this condition. The development of novel vaccines and explore emerging therapeutic approaches, such as tailoring novel antiviral combinations, immunomodulatory treatments, and even targeted therapies for the elderly population is imperative. These measures should be complemented by strategies to increase vaccination uptake and adherence to public health guidelines, particularly in high-risk groups. Furthermore, the exploration of precision medicine approaches offers hope for personalized treatment plans that can meet the unique needs of individual patients. In summary, the aging population poses significant challenges in the context of viral diseases. By deepening our understanding of the complex interplay among aging, immunity, and viral pathogens, we can strive to develop more effective prevention, diagnostic, and treatment strategies. This will not only improve the health status of the elderly but also enhance the overall resilience of our society in the face of emerging infectious diseases.

## Figures and Tables

**Table 1:** Comparison of SARS-CoV-2 and influenza viruses and the mechanism of their coinfections.

Virus	SARS-CoV-2	Influenza Virus	Reference
**Nucleic acid**	Single-stranded Positive-sense RNA virus	Single-stranded Negative-sense RNAvirus	
**Infection target**	S protein and ACE2	HA protein and Sia	
**Coinfection mechanism**	IAV infection induces elevated ACE2 expression		[8]
	Coinfection increases the viral load of SARS-CoV-2 and influenza		[8,45]
	Coinfection extends the duration of the initial viral infection		[46]
	Coinfection leads to heightened immune cell infiltration		[46]
	Coinfection leads to severe lymphopenia in peripheral blood		[47]

**Table 2:** Clinical manifestations and complications of coinfection with influenza and COVID-19 in the elderly.

**Neuropsychiatric system**	Seizures and meningoencephalitis	[111,112]
	Encephalopathy, encephalitis, transverse myelitis, stroke, and dementia	[4,113,114]
	Delirium, headache, and dizziness	[109,111,115]
	Anosmia, ageusia, and fatigue	[109,116]
	Illusion and brain fog	[4,113,114]
	Cognitive and motor impairment	[117,118]
	Guillain–Barré and Reye syndrome	[119,120,121,122]
**Respiratory system**	Acute respiratory distress syndrome (ARDS)	[40,110]
	Pneumonia and hypoxemia	[40,123]
	Respiratory failure/acidosis/alkalosis	[107,124,125,126]
	Pulmonary fibrosis/lymphadenopathy, pleural effusions, and linear atelectasis	[36,40]
	Secondary bacterial pneumonia	[7]
	Cough, dyspnea, nasal congestion, pharyngalgia, expectoration, and runny nose	[36,40,109,127]
**Cardiovascular system**	Cardiopulmonary arrest	[40,112,128]
	Acute cardiac injury and fulminant myocarditis	[40,110,128]
	Acute heart failure and coagulation disorder	[7,129]
	Chest distress/chest pain	[110,130]
**Urinary system**	Acute kidney injury (AKI) and anuric	[7,110,131]
	Metabolic acidosis and metabolic alkalosis	[132,133]
	Electrolyte disturbances	[133,134]
**Digestive system**	Acute liver injury and hepatic dysfunction	[40,110,135]
	Diarrhea and nausea	[136]
	Loss of appetite and vomiting	[115]
**Others**	Cytokine storm	[110]
	Viral sepsis and sepsis	[137,138]
	Dehydration and exacerbation of chronic disease	[4]

**Table 3:** **Current mainstream** combination vaccines combating SARS-CoV-2 and influenza.

Platform	Name	Vaccine Effectiveness	Reference
**mRNA vaccines**	AR-CoV/IAV	HAI Ab (>1: 1024) SARS-CoV-2 NAb (>1: 849) After 2 doses	[203]
	FLUCOV-10	Provided complete protection to immunized mice	[201]
**Recombinant protein vaccines**	FLU-COVID	As efficient as influenza or SARS-CoV-2 mono vaccines	[204]
	-	High level IgG, HAI Ab High titers NAb against SARS-CoV-2 Omicron BA.5	[205]
	-	HAI Ab (>1: 1024) Long-lasting and high-titer Nabs against SARS-CoV-2	[206]
	-	Broad NAbs against VOCs Cross-protection against IAVs	[207]
**Virus-like-particle vaccine**	VLP-RBD-GM-CSF-IL-12	Long-lasting and high-titer Nabs against H1N1 Abs against ACE2 binding to RBD	[208]
**Influenza-based vaccines**	TM-RBD-HA	Elicited Nabs and provided protection with IAV and SARS-CoV-2 in mice.	[209]
	Pneucolin (dNS1-RBD)	Exhibited great safety and efficacy in the elderly	[210]
	Flu-RBD	High titers of HA-specific and RBD-specific IgG Abs	[211]
	ΔNA(RBD)-Flu	A single dose can generate equivalent Nab titers to those produced by two doses of mRNA vaccines.	[212]
**VSV-based vaccines**	-	Effectively protected hamsters or mice against SARS-CoV-2 Delta, H1N1, and H3N2	[213]
**Adenoviral vectors vaccines**	Adc68-CoV/Flu	Effectively induced SARS-CoV-2-targeting Abs and anti-influenza Abs in mice	[214]

## Data Availability

The data presented in this study are available upon request from the corresponding author.

## Abbreviations

The following abbreviations are used in this manuscript:

SARS-CoV-2	Severe acute respiratory syndrome coronavirus 2
CFR	case fatality ratio
IAV	influenza A virus
IBV	influenza B virus
HA	hemagglutinin
NA	neuraminidase
SASP	senescence-associated secretory phenotype
HSCs	hematopoietic stem cells
ICU	intensive care unit
ETC	electron transport chain
HPA	hypothalamic–pituitary–adrenal
HSCs	hematopoietic stem cells
ICU	intensive care unit
ETC	electron transport chain
HPA	hypothalamic–pituitary–adrenal
TF	tissue factor
ALT	alanine aminotransferase
AST	aspartate aminotransferase
GGT	gamma glutamyl transferase
LDH	lactate dehydrogenase
COVID-19	Coronavirus Disease 2019
VOCs	variants of concern
ACE2	angiotensin-converting enzyme 2
RBD	receptor-binding domain
SARS	severe acute respiratory syndrome
RAS	renin–angiotensin System
ARDS	acute respiratory distress syndrome
AKI	acute kidney injury
Ang II	angiotensin II
vWF	von Willebrand factor
WHO	World Health Organization
NPIs	nonpharmaceutical interventions
PCR	polymerase chain reaction
Ct	cycle threshold
IL-1	interleukin-1
IL-6	interleukin-6
TNF-α	tumor necrosis factor-alpha
ER	endoplasmic reticulum
PERK	protein kinase R-like endoplasmic reticulum kinase
eIF2α	eukaryotic initiation factor 2 alpha
ATF-4	activating transcription factor 4
ATF-6	activating transcription factor 6
CHOP	C/EBP homologous protein
S1P	serineproteasesite-1
S2P	serine 534 proteasesite-2
IRE1	inositol-requiringenzyme1
JNK	c-Jun N-terminal kinase
BBB	blood–brain barrier
CK	creatine kinase
CK-MB	creatine kinase isoenzyme MB
cTnI	cardiac troponin I
BNP	brain natriuretic peptide
PT	prothrombin time
APTT	activated partial thromboplastin time
ACS	acute coronary syndrome
MMP-13	matrix metalloproteinase-13
DCs	dendritic cells
APCs	antigen-presenting cells
MHC-II	MHC class II molecules
NK	natural killer
ABCs	age-associated B cells
TCR	T cell receptor
Treg	regulatory T cell
Teff	effector T cell
Th17	T helper17
BMI	body mass index
CARS	anti-inflammatory response syndrome
UPR	Unfolded Protein Response
CNS	central nervous system
VLP	virus-like particle
M2e	matrix 2 ectodomain of the influenza virus
